# Eco-friendly fish gelatin edible films enhanced with mango peel extract and fatty acid sucrose esters for sustainable fruit packaging

**DOI:** 10.1007/s13197-025-06476-9

**Published:** 2025-11-10

**Authors:** Farah Faiqah Fazial, Ong Ying Ying, Azfaralariff Ahmad

**Affiliations:** 1 Faculty of Chemical Engineering & Technology, University Malaysia Perlis (UNIMAP), UNICITI Alam Campus, Sungai Chuchuh, 02100 Padang Besar, Perlis Malaysia; 2 Food Technology Division, School of Industrial Technology, Universiti Sains Malaysia, 11800 Gelugor, Penang Malaysia

**Keywords:** Fish gelatin, Edible film, Mango peel extract, Fatty acid sucrose ester, Food packaging, Cherry tomatoes

## Abstract

New consumer trends demand sustainable packaging solutions to extend fruit shelf life. In this study, mango peel extract (MPE), rich in polyphenols and antioxidants, and hydrophobic fatty acid sucrose esters (FASE) were incorporated into fish gelatin film packaging for cherry tomatoes to extend shelf life and reduce environmental impact from fruit spoilage. MPE extracted with 80% ethanol showed the highest total phenolic content (TPC) of 72.64 mg/g and antioxidant activity of 76.76%. A 3% concentration of 80% ethanolic MPE was used in the film forming solution. FASE (5%, 10%, and 15% w/w polymer) improved water barrier properties (from 1.02 to 0.97 g mm/m^2^ h Pa) and decreased tensile strength (TS) (from 257.93 to 84.73 MPa) of the fish gelatin film. The film containing 10% FASE (FL10) effectively slowed weight loss, minimized color changes, and maintained the firmness of cherry tomatoes compared to the control. This study highlights the potential of bioactive, biodegradable films as a sustainable alternative to synthetic packaging for fruit preservation.

## Research highlights

1. Fish gelatin film was successfully incorporated with mango peel extract (MPE) and fatty acid sucrose ester (FASE).
2. Water barrier properties of fish gelatin film improved as concentration of fatty acid sucrose ester (FASE) increased.
3. Fish gelatin film with 10% FASE showed improvement in increasing shelf life of cherry tomatoes.

## Introduction

Considering the effect of municipal solid waste on our planet, many studies and research have been undertaken to explore their roles in reducing and replacing the use of non-biodegradable solid waste. Furthermore, food packaging constitutes a significant portion of litter that frequently accumulates on beaches and in waterways. This situation poses a serious threat to aquatic wildlife and birds, which may ingest plastic packaging and debris, endangering their lives (Roman et al. 2022).

Edible films and coatings enhance food quality, shelf life, safety, and functionality by preventing physical, chemical, and microbiological contamination. Composed of polymers providing mechanical strength, these thin layers depend significantly on the inherent properties of film-forming materials such as proteins, carbohydrates, and lipids (de Vargas et al. 2022). Combining edible films with antioxidants like vitamins C and E, selenium, and carotenoids such as beta-carotene, lycopene, lutein, and zeaxanthin can enhance their oxidation-inhibiting properties. Mango peel is rich in polyphenols, anthocyanins, and carotenoids, demonstrating strong antioxidant activity (Marcillo-Parra et al. 2021).

The use of fatty acid sucrose ester (FASE) as an edible film component has been explored for a wide range of applications. Its incorporation into various natural polymeric matrices has been reported, including fish gelatine, arabinoxylan, pullulan, hydroxypropyl methylcellulose, and a combination of methylcellulose, glucomannan, and pectin (Amalini et al. 2018). FASE with an HLB value of 5 are effective in emulsifying water-in-oil. Low HLB makes them excellent film coating agents for preserving tomatoes, as they regulate water loss, decrease respiration rate, and maintain produce quality during storage (Zhao et al. 2023). They can regulate water loss decrease respiration rate and maintain the quality of the produce during storage. The hydrophobic moiety of FASE can act as a barrier against water vapor transmission and provide resistance to vapor and gas transport. SE incorporated in rice starch film has been developed to extend the postharvest ripening of Cavendish bananas at 20 °C, showing positive impacts in terms of reduced weight loss, prolonged shelf life up to 12 days, and delayed chlorophyll degradation (Thakur et al. 2019). Another study on chicken packaging found that adding fatty acids (10–18 carbons) to film packaging improved the barrier against water and light, as well as slowed down the degradation of vitamin C (Wu et al. 2023).

Cherry tomatoes are an ideal focus for this study due to their high perishability, significant economic value, and nutritional importance. With their thin skin and high-water content, cherry tomatoes are particularly susceptible to physical damage, microbial spoilage, and moisture loss, making them challenging to preserve during storage and transportation. Their rich content of antioxidants like lycopene, vitamins A, C, and E, and phenolic compounds enhances their appeal as a nutritious and functional food, but these qualities are quickly lost without proper postharvest management. Additionally, their small size and delicate texture increase the risk of fungal infections, such as *Botrytis cinerea*, especially in humid conditions, leading to rapid quality deterioration and reduced marketability.

Globally, postharvest losses in fruits and vegetables can reach up to 45% of total production, with tomatoes often accounting for 12–30% of these losses, depending on the region and storage infrastructure (Thrikawala et al. 2025). In developing countries, where cold chain systems are limited, these losses can severely impact food security and farmer livelihoods. Implementing hydrophobic packaging solutions tailored to cherry tomatoes offers a strategic approach to mitigating moisture-induced spoilage, extending shelf life, and reducing food waste. Such innovations not only enhance the economic sustainability of the supply chain but also promote environmental sustainability by replacing synthetic plastics with biodegradable edible films (Korte et al. 2023).

The selection of *Moringa oleifera* extract (MPE) and fatty acid sucrose ester (FASE) was based on their demonstrated bioactive and functional properties. MPE is rich in antioxidants like polyphenols and flavonoids, offering strong free radical scavenging and antimicrobial activity, which are crucial for food preservation. FASE enhances the hydrophobicity of film matrices, improving water vapor barrier properties. Previous studies have separately investigated MPE for its antioxidant and antimicrobial properties and FASE for their hydrophobicity and barrier function. This research demonstrates their synergistic effect on both functional and structural properties, which has not been widely explored in previous studies. Their combined use in a fish gelatin matrix addresses key challenges in creating sustainable and functional packaging.

Therefore, this innovative approach of incorporating FASE into fish gelatine film can enhance the shelf life of cherry tomatoes by providing moisture and gas barriers, preventing dehydration, and slowing down oxidative processes that affect flavour and colour. Additionally, MPE/FASE-containing films may help preserve the flavour and aroma of tomatoes by minimizing exposure to external odours and flavours.

## Experimental

### Reagents and solutions

Glycerol, Distilled water, Ethanol, 2 phenl-1,1-hydrazine (DPPH), Folin–Ciocalteu reagent, Sodium Carbonate anhydrous, Gallic acid, Hydrochloric acid, Fatty acid sucrose esters was obtained from MERCK.

### Preparation of mango peel extract (MPE)

The mango was obtained from local farmer at Kangar market in Perlis, Malaysia. The peels were first removed, washed with tap water, then rinsed with distilled water. After drying in a food dehydrator (Parallex, USA) for 24 h at 50 °C, they were ground into powder using an electric blender (Waring, USA). The powdered peels were stored in an air-tight container in a cool, dark place at − 18 °C until needed. The ground mango peel (20 g) was immersed in ethanol at different concentrations (40%, 60%, 80%) in conical flasks. These flasks were then placed in a water bath (Memmert, Germany) and shaken at 100 rpm at room temperature for 2.5 h. Afterward, the samples were centrifuged at 8000 rpm for 15 min to separate the extract. The residues were then re-immersed for a second extraction to enhance extraction efficiency.

### Total phenolic content of MPE

The 0.3 ml of MPE was mixed with 2.5 ml of 10% Folin–Ciocalteu reagent followed by addition of 2 ml of 7.5% sodium carbonate solution. The mixture was measured by a UV-160 spectrophotometer (Shimadzu, Japan) after incubated at 50 °C for 50 min at 760 nm (Souli et al. 2022).

### Antioxidant activity of MPE

The ability of scavenging free radicals were correlated with the total of phenolic contents in the films. Initially, 3 ml of the MPE extract was mixed with 1 ml of 0.1 mM of ethanolic solution of 2-phenl-1,1-hydrazine (DPPH). Then, the mixture was placed and incubated in dark condition for 30 min. The absorbance was measured at 517 nm by using UV–Vis spectrophotometer (model UV-160, Shimadzu, Kyoto, Japan) (Souli et al. 2022). The percentage of DPPH scavenging was determined by the following equation:

$$ DPPH\,scavenging\,activity (\% ) = \frac{Abs DPPH - Abs extract}{{Abs DPPH }} \times 100 $$

where Absorbance DPPH indicated the absorbance of the ethanolic DPPH solution and Abs extract indicated the absorbance of the extract of the sample (Susmitha et al. 2021).

### Preparation of film packaging solution and its casting method

The film-packaging solution, 20% fish gelatin was prepared. Fish gelatin powder was added in 60 °C of distilled water and stirred continuously for 30 min. Afterward, the solutions were heated to a constant temperature with the addition of 30% glycerol (w/w fish gelatin) and selected MPE extract having high concentration of total phenolic content and antioxidant capacity. Subsequently, different concentrations of fatty acid sucrose ester were added in the solution. Finally, 80 g aliquots of the film-forming solutions were distributed onto rimmed plastic plates and dried for 24 h at room temperature (Susmitha et al. 2021). The resulting films were then evaluated for their mechanical and barrier properties.

### Tensile strength (TS) and elongation at break (EAB)

The TS and EAB test were conducted by following the standard method from ASTM Standard (D882-02) using the TA.XT-Plus texture analyser (Stable Micro System, Surrey, UK) equipped with tensile load cell of 5 kg at a speed of 0.5 mm/s. Five measurements for each sample (2.5 × 10 cm^2^) with the initial grip length of 2.5 cm were used for testing (Susmitha et al. 2021).

### Water vapor permeability

WVP was determined per ASTM E96-00 using gas permeation cells (4.5 cm diameter, 2.8 cm height). Glass cells with 25 g silica gel (0% RH) were oven-dried at 120 °C for 24 h. Test films were sealed on top and placed in a 30 °C desiccator with distilled water (100% RH) for 7 days. Weighing of the glass cells was conducted every 24 h, recorded to 0.0001 g, and plotted over time. Linear regression (r^2 ≥ 0.99) calculated each slope. The measurement of WVP was determined by using the equation below:

$$ WVP = \frac{WVTR \times L}{{\Delta P}} $$

where WVTR is the water vapor transmission rate (g m^−2^ h^−1^) through a film which is calculated from the slope of the straight line that divided by the exposed film area (m^2^) Meanwhile, L is the mean film thickness (mm) and ∆P is the partial water vapor pressure difference (Pa) from the two sides of the film. The WVP measurements was done in triplicates for each batch of films (Harrazi et al. 2024).

### Light barrier properties

The measurement of the light barrier properties of the films was done by exposing the films to light absorption at wavelength 550 nm. By placing a rectangular film sample into a spectrophotometer, the film transparency was measured. The spectrophotometer used to record the absorbance is UV-160A, UV–Vis spectrophotometer (model UV-160, Shimadzu, Kyoto, Japan). The equation to calculate the transparency of the film are as followed:

$$ T = \frac{{A_{600} }}{x} $$

where A550 is the absorbance at 550 nm while x is the film thickness (mm). From this equation, the higher the value of T, the lower the degree of transparency. The transparency test was done in triplicates for each batch of films (Ye et al. 2022).

### Analysis on cherry tomatoes wrapped with prepared film

The tomatoes were washed, dried, and then wrapped with the film prepared from a selected formulation: Selected fish gelatin film having excellent mechanical properties, barrier characteristics, and high antioxidant capacity was used in this study. The wrapped tomatoes were refrigerated at 4 °C for 10 days. Parameters including color, weight loss, and texture were assessed from day 0 and every 2 days thereafter (Days 2, 4, 6, 8, and 10), and compared with the initial conditions of the unwrapped tomato.

### Colour

The redness of the cherry tomato was measured by using a colorimeter and calibrated by white and black tile. Cherry tomatoes in this study were at the light red stage of maturity. Tomatoes at the light red stage are often used in commercial supply chains, as they strike a balance between marketability and transport durability. Hence, studying this stage ensures the findings are directly relevant to real-world applications. Each cherry tomato sample was measured six times. Redness of the cherry tomato is an important parameter in determining the level of maturity as a fruit that is rich of lycopene (Mukhopadhyay et al. 2023).

### Weight loss

Weighing method was used to measure weight loss. The same cherry tomatoes were weighed from the first day to the end of the experiment and the weight reading were taken every 2 days during storage. Percentage loss of the initial total weight was expressed as the result of weight loss. Initial weight before storage (W_o_) was compared with the final weight after storage in the period to observe the differences. The percentage of weight loss was calculated using the following equation:

$$ Weight\,Loss(\% ) = \frac{{W_{f} - W_{o} }}{{W_{o} }} $$

### Firmness

A texture analyser was used to determine the firmness of the tomatoes by the compression force. The tests were conducted with a 50 mm diameter cylindrical probe and a 50 N load. The test speed set was1 mm/s, 2 mm/s for pre-test and 5 mm/s for post-test accordingly. The results were expressed in Newton (da Costa de Quadros et al. 2020).

### Statistical analysis

SPSS 26.0 was used in this study to analyse the data. Data was subjected to one-way variance analysis by using Duncan’s test with confidence level as *p* ≤ 0.05.

## Results and discussion

### Total phenolic content and antioxidant capacity of mango peel extract (MPE)

**Fig. 1:**
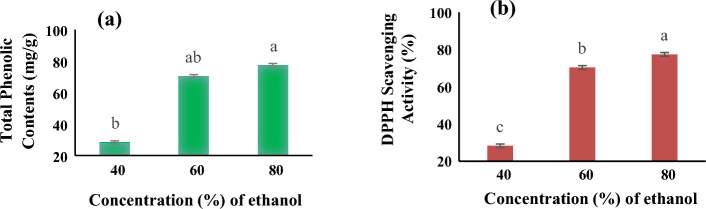
Total phenolic content (mg/g) (**a**) and DPPH scavenging activity (**b**) of MPE by different concentrations of ethanol

Total phenolic content (TPC) was assessed using Folin–Ciocalteu Reagent (FCR) with tungsten and molybdenum, measuring absorbance at 760 nm. TPC values from MPE extracted with 40%, 60%, and 80% ethanol were 25.45 mg/g, 62.70 mg/g, and 72.64 mg/g, respectively (Fig. 1a). Extraction using 80% ethanol showed the highest TPC, indicating superior bioactivity. Phenolic compounds are a group of secondary metabolites which are soluble in organic solvent like ethanol. Higher concentration of ethanol can enhance the solubility of phenolic compound leading to greater yield (Zhang et al. 2022). Since phenolic compounds exhibit a variety of biological activities, they can exhibit excellent antioxidant activities by scavenging free radicals and reduce oxidative stress that linked to various diseases. Figure 1b supports the theory whereby the DPPH scavenging activity was measured at 26.62%, 70.77%, and 76.76%, respectively. Lower absorbance values indicate more diluted MPE solutions with higher transmittance, reflecting stronger antioxidant activity. This suggests that MPE extracted with higher ethanol concentrations (80%) exhibited greater ability to scavenge free radicals and showed more pronounced discoloration compared to those extracted with 40% and 60% ethanol.

Among the solvents tested (40% to 80% ethanol), 80% ethanol yielded the highest extraction efficiency, with TPC at 0.3064 mg/g and antioxidant activity at 76.76%. Therefore, MPE extracted with 80% ethanol was chosen for further analysis and application in films.

### Analysis of fish gelatin film containing different concentration of fatty acid sucrose ester (FASE)

**Fig. 2:**
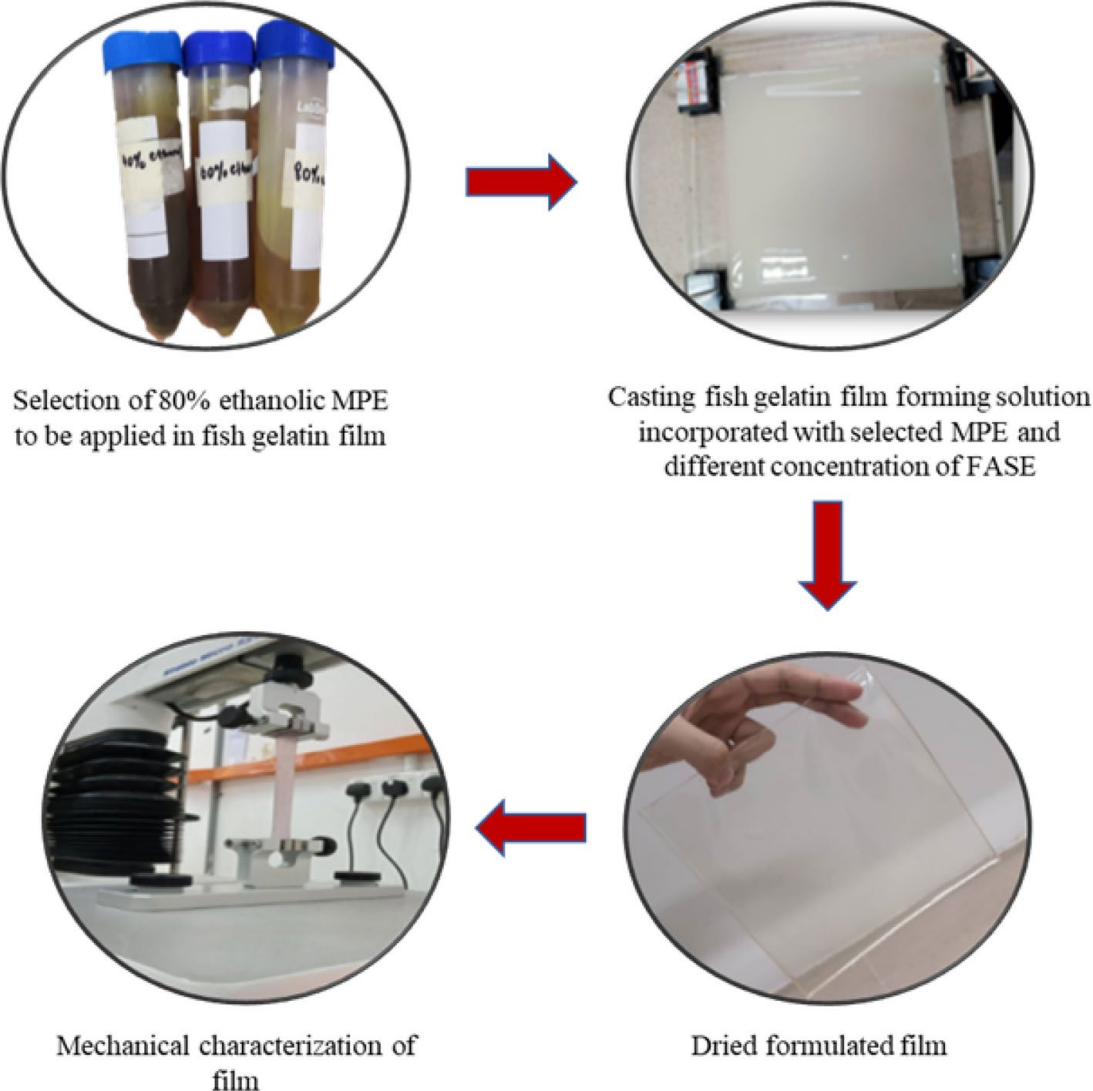
Schematic diagram of the method for preparing fish gelatin film containing mango peel extract (MPE) and fatty acid sucrose ester (FASE) before film analysis was conducted

The fish gelatin film-forming solution, incorporated with 80% ethanolic MPE and different concentrations of fatty acid sucrose ester (FASE), was prepared before the casting technique. Further drying was conducted to obtain the dried film for analysis, as illustrated in Fig. 2.

#### Mechanical properties of film as affected by different concentrations of fatty acid sucrose ester (FASE)

Edible films require an understanding of mechanical properties like tensile strength (TS), elongation at break (EAB), and Young’s modulus (E) which vary with film composition (Shah et al. 2023). Table 1 shows TS, EAB, and E values for films with different sucrose ester fatty acid (FASE) concentrations. Higher FA concentrations led to lower TS, EAB, and E values; TS decreased notably from 257.93 to 84.73 MPa, EAB decreased from 170.81 to 127.87%, and E reduced from 1.46 to 0.15 MPa. This result may be attributed to the uniform dispersion of FASE droplets within the film network, which reduces protein–protein interactions. The addition of fatty acids typically inhibits protein–protein interactions in the film network, resulting in discontinuities in the film matrix (Pirsa and Aghbolagh Sharifi 2020). The hydrophobic portion of the FASE was exposed to the protein-rich phase that reduce the protein–protein interaction thus lowering the elasticity. Hence, a lower EAB was recorded when higher amount of FA incorporated (*p* < 0.05). The increased mobility among short chain fatty acid may also contribute to a further reduction in E as the polymer chains can move more freely under stress (Eslami et al. 2023).

**Table 1:** Mechanical properties of fish gelatin film incorporated with different concentration of FASE at 5%, 10% and 15%

Sample	Concentration of FASE (%, w/w polymer)	Tensile strength (MPa)	Elongation at break (%)	Young’s Modulus (MPa)
FL5	5	257.93 ± 10.12^a^	170.81 ± 8.79^a^	1.46 ± 0.02^a^
FL10	10	184.53 ± 63.72^b^	165.96 ± 8.48^a^	1.08 ± 0.27^b^
FL15	15	84.73 ± 6.59^c^	127.87 ± 8.48^b^	0.51 ± 0.06^c^

Different superscript within the same column indicates significant differences (*p* ≤ 0.05). FL5, FL10, and FL15 is Fish gelatin film with 15, 10, and 15% of fatty acid

#### Water vapor permeability (WVP) of film affected by different concentration of FASE

**Fig. 3:**
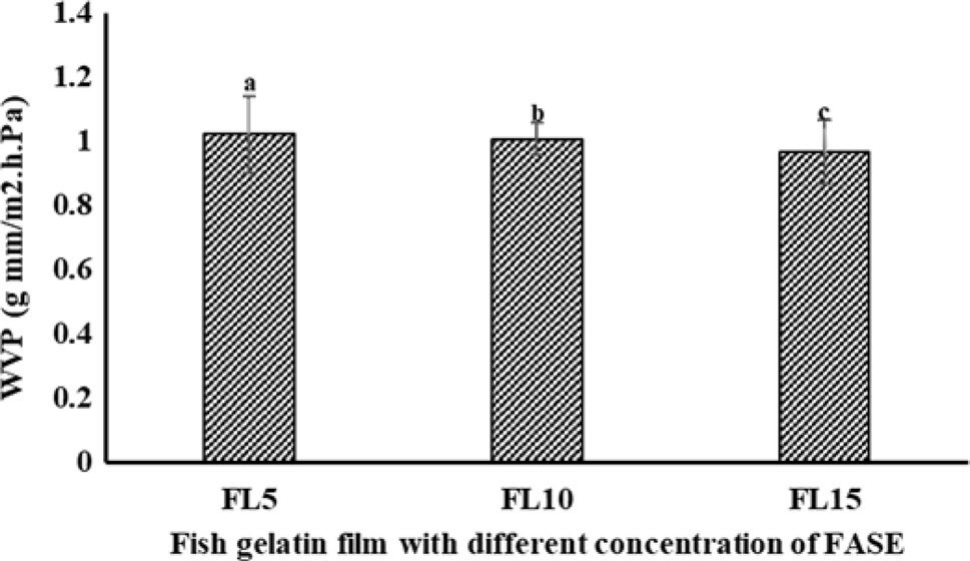
WVP of fish gelatin film incorporated with different concentration of FASE at 5%, 10% and 15%. FL5, FL10, and FL15 is Fish gelatin film with 15, 10, and 15% of FASE. Different superscript indicates significant differences (*p* ≤ 0.05)

The WVP of the film with the addition of varying concentrations of FASE was depicted in Fig. 3. Moisture transfer between food and its surroundings is critical in food packaging films, influencing product shelf life. The results indicate a decreasing trend in WVP from 1.02 to 0.97 g mm/m^2^·h·Pa with increasing FASE content. The addition of hydrophobic FASE likely induces interactions between biopolymers and FASE within the film composition. Additionally, the chain length significantly affects FASE hydrophobicity, with longer chains like lauric acid (LA) (C_12_), palmitic acid (PA) (C_16_), and stearic acid (SA) (C_18_) showing varying degrees of hydrophobicity. The observed decrease in WVP for LA, PA, and SA films can thus be attributed to the chain length of the FASE. In addition to chain length, factors like saturation, polarity, and branching also affect film WVP. Longer, non-polar, saturated, and linear lipid molecules create films that are more rigid, cohesive, and less permeable compared to shorter, unsaturated chains with higher branching (Shahidi and Hossain 2022). FA combines hydrophilic and hydrophobic traits, facilitating dispersion within the film matrix and interaction with protein chains via hydrogen bonding to the protein molecule’s hydrophilic head (Li et al. 2023).

#### Light barrier properties affected by FASE

Table 2 presents light transmission and transparency values for fish gelatin films incorporating different concentrations of FASE. Higher percentages of FASE from 5 to 15% led to lower light transmission, indicating better light barrier properties. Conversely, increased FASE content resulted in higher opacity ranging from 1.08 to 2.66. The rise in fatty acid concentration in the film-forming solution reduced water absorption and lowered film water content, influencing transparency due to water and crystalline content effects (Yang et al. 2023). Different FASE chain lengths aligned differently in the film matrix, affecting film characteristics such as transmission. Based on the result, the amounts of lipids used highly correlated with transmission of films for UV and visible light ranges. In terms of specific applications, FASE are often utilized for their emulsifying properties and can affect the mechanical and barrier properties of films as packaging materials. The altered light transmission characteristics can be significant in food packaging and other applications where opacity and light permeability are important (Teng et al. 2021).

**Table 2:** Light transmittance and transparency value of fish gelatin film incorporated with different concentrations of FASE (5%, 10%, 15%)

Sample	Light transmittance (%)						Opacity at T_600_
	200	400	500	600	700	800	
FL5	8.05 ± 0.05^c^	2.05 ± 0.01^f^	3.52 ± 0.01^e^	6.65 ± 0.01^e^	8.41 ± 0.04^b^	10.81 ± 0.02^a^	1.08 ± 0.02^b^
FL10	6.41 ± 0.01f	9.67 ± 0.03e	9.67 ± 0.01d	12.15 ± 0.01c	16.95 ± 0.02b	17.20 ± 0.01a	1.13 ± 0.01^b^
FL15	5.72 ± 0.01^f^	9.67 ± 0.01^e^	12.16 ± 0.01^d^	14.62 ± 0.01^c^	15.75 ± 0.07^b^	17.21 ± 0.01^a^	2.66 ± 0.07^a^

Different superscript within the same column indicates significant differences (*p* ≤ 0.05). FL5, FL10, and FL15 is fish gelatin film with 15, 10, and 15% of FASE

### Analysis on cherry tomato wrapped with selected film

Based on the results of film characteristics in Sect. 3.2, FL10 was selected to be further investigated for its application in fruit packaging, in this case cherry tomatoes were chosen. FL10 has excellent mechanical properties (184.53 MPa), better resistance towards water (1.05 g mm/m^2^ h Pa), and excellent transparency (1.13).

**Fig. 4:**
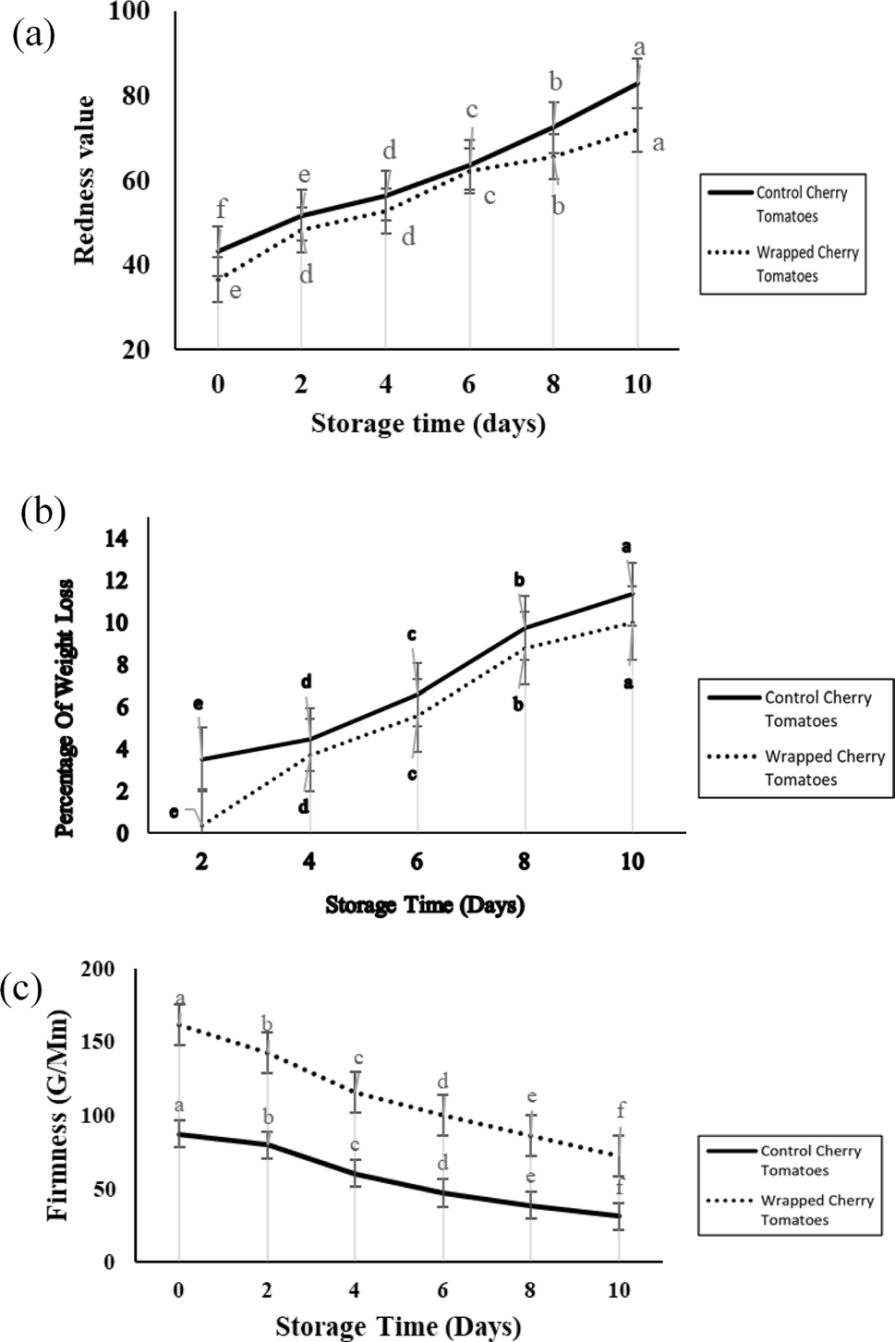
Measurement of redness (**a**), weight loss (**b**), and firmness (**c**) of both control and FL10 film wrapped cherry tomatoes during storage at 4 °C for 10 days. Different superscript indicates significant differences (*p* ≤ 0.05)

Figure 4a illustrates the color changes observed in control and FL10 film-wrapped cherry tomatoes during a 10-day storage period. The surface color changes are attributed to various pigmentations present. Cherry tomatoes typically exhibit a vibrant red color, indicating their maturity and high antioxidant lycopene content. According to colorimeter readings, negative values of a* indicate green hues, whereas positive values denote red hues. Based on the results, the a* values of wrapped cherry tomatoes were compared with the control over a 10-day storage period. The red color intensity of the control tomatoes increased more significantly compared to the wrapped ones. Specifically, the a* value for the control tomatoes increased from 43.2 to 83%, while for the wrapped tomatoes, it rose from 36.87 to 71.67%. The varying development of red color in each cherry tomato is attributed to their different maturity levels before the experiment. The study provides evidence that the film forms a protective barrier, effectively reducing lycopene oxidation compared to the control (Li et al. 2023). Edible coatings can minimize the dehydration and oxidation of food products, preserving taste, texture, and color (Matloob et al. 2023).

Percentage of weight loss of the cherry tomatoes was presented in Fig. 4b. The weight loss of cherry tomatoes increased over storage time. According to the result, the percentage of weight loss of the control was higher than the wrapped cherry tomatoes. Weight loss of control and wrapped cherry tomatoes increased to 11.6929% and 8.4331% respectively. Transpiration in cherry tomatoes is linked to the water vapor pressure of the surrounding atmosphere and the tomato surface. Conversely, weight loss due to respiration occurs as carbon atoms are released in the form of carbon dioxide molecules from the tomato surface (Setyorini 2021). In addition, the weight loss of the fruit results from metabolic degradation in the cell wall, leading to a decrease in water retention capacity observed during senescence (Saleem et al. 2023).

Firmness reduction often attributed to different factors such as loss of cell turgor pressure and cell wall and polysaccharides degradation (He et al. 2022). Edible film significantly (*p* < 0.05) reduced the firmness of cherry tomatoes stored at 4 °C (Aragüez et al. 2020). Figure 4c shows firmness loss over time, with initial values of 87.71 g/mm for control and 73.85 g/mm for FL 10 film-wrapped tomatoes. After 10 days, firmness decreased to 31.93 g/mm for control and 40.71 g/mm for FL 10 wrapped tomatoes, indicating a less intense firmness loss of 33.14 g/mm compared to the control. As compared to the control, the firmness loss for FL10 film wrapped cherry tomatoes was less intense with difference of 33.14 g/mm from the first day of storage. The applied film effectively extended the postharvest life of cherry tomatoes by preserving their quality, as evidenced by slowed maturation observed in terms of reduced weight loss, color changes, and firmness (Roshandel-hesari et al. 2022). The firmness loss in cherry tomatoes during storage primarily results from the ripening process, involving starch breakdown into sugars due to the structural role of starch granules in plant cells. This ripening process leads to continuous firmness reduction through transpiration-induced moisture loss and enzymatic degradation of the tomato cell wall. Specifically, pectic enzymes and cellulose activity play crucial roles in softening and firmness reduction (Saleem et al. 2023).

## Conclusions

Fish gelatin edible film incorporated with mango peel extract (MPE) and FASE was prepared and evaluated based on mechanical, water barrier, and light barrier properties. Initially, highest total phenolic content and antioxidant capacity from 80% ethanol MPE extract was incorporated into the film to provide protection of cherry tomatoes against oxidation. The incorporation of FASE at concentrations ranging from 5 to 15% w/w of the polymer resulted in a reduction in mechanical properties but significantly improved water vapor permeability performance. The high range tensile strength (TS) and strong barrier against water molecules has good potential in preserving fruits from various physical and microbial issues that compromise their quality and edibility. The fish gelatin film containing 10% FASE (FL10) was selected as it demonstrated high TS, strong barrier against water, and transparence film show significant improvement in the packaging of cherry tomatoes. FL 10 wrapped cherry tomatoes resulted in preserving redness value (*a), reducing water loss, and maintaining the firmness by slowing the rate of fruit spoilage. This study uniquely highlights the effectiveness of FASE-enhanced films in maintaining the quality of cherry tomatoes by reducing weight loss, preserving firmness, and slowing spoilage. By demonstrating the real-world benefits of FASE in edible packaging, this research expands current understanding of hydrophobic emulsifiers in food preservation. The findings support the development of sustainable packaging solutions that reduce reliance on synthetic plastics, contributing to environmentally friendly postharvest technologies. Further studies are recommended to evaluate its performance with other perishable fruits under varying environmental conditions could provide broader insights into its potential as a sustainable and versatile food packaging solution.

## Data Availability

The data that support the findings of this study are available from the corresponding author, (Farah Faiqah Fazial) upon reasonable request.

## Abbreviations

FASE: Fatty acid sucrose ester
MPE: Mango peel extract
TS: Tensile strength
EAB: Elongation at break
TPC: Total phenolic content
DPPH: 2,2-Diphenyl-1-picrylhydrazyl
